# Biogas production from food waste via co-digestion and digestion- effects on performance and microbial ecology

**DOI:** 10.1038/s41598-017-15784-w

**Published:** 2017-12-15

**Authors:** Mirzaman Zamanzadeh, Live Heldal Hagen, Kine Svensson, Roar Linjordet, Svein Jarle Horn

**Affiliations:** 1 Faculty of Chemistry, Biotechnology and Food Science, Norwegian University of Life Sciences, P. O. Box 5003, N-1432 Ås, Norway; 2 Department of Environmental Health Engineering, School of Public Health, Tehran University of Medical Sciences, P.O. Box 14155-6446 Tehran, Iran; 3 Department of Civil and Environmental Engineering, University of Waterloo, 200 University Avenue West, Waterloo, Ontario, N2L 3G1 Canada; 4 Nibio, Norwegian Institute of Bioeconomy Research, Ås, Norway

## Abstract

In this work, performance and microbial structure of a digestion (food waste-only) and a co-digestion process (mixture of cow manure and food waste) were studied at mesophilic (37 °C) and thermophilic (55 °C) temperatures. The highest methane yield (480 mL/g VS) was observed in the mesophilic digester (MDi) fed with food waste alone. The mesophilic co-digestion of food waste and manure (McoDi) yielded 26% more methane than the sum of individual digestions of manure and food waste. The main volatile fatty acid (VFA) in the mesophilic systems was acetate, averaging 93 and 172 mg/L for McoDi and MDi, respectively. Acetate (2150 mg/L) and propionate (833 mg/L) were the main VFAs in the thermophilic digester (TDi), while propionate (163 mg/L) was the major VFA in the thermophilic co-digester (TcoDi). The dominant bacteria in MDi was *Chloroflexi* (54%), while *Firmicutes* was dominant in McoDi (60%). For the mesophilic reactors, the dominant archaea was *Methanosaeta* in MDi, while *Methanobacterium* and *Methanosaeta* had similar abundance in McoDi. In the thermophilic systems, the dominant bacteria were *Thermotogae, Firmicutes* and *Synergistetes* in both digesters, however, the relative abundance of these phyla were different. For archaea, the genus *Methanothermobacter* were entirely dominant in both TDi and TcoDi.

## Introduction

Anaerobic digestion process has widely been employed for treatment of various organic wastes because the process can be used for production of value-added products such as an energy-rich gas and bio-fertilizer. This process is carried out by a complex microbial community which degrade various organic compounds into final products such as methane and carbon dioxide, collectively called biogas.

There are presently many research efforts worldwide on anaerobic digestion of food waste to improve process efficiency, stability and economic competitiveness. Studies of co-digestion of food waste generally found that inclusion of food waste was beneficial for methane yield^1–3^, while digestion processes with food waste as the sole substrate were often found to be unstable^3–5^. Several researchers have reported the benefits of using mixed feedstocks, including increased biogas production, enhanced degradation rates and higher digester capacity^1,6,7^. The beneficial effects of co-digestion are mostly related to a balanced availability of macro- and micronutrient required by the microbial community, optimal moisture content, buffer capacity and dilution of inhibitory or toxic compounds. Additionally, co-digestion may improve the process kinetics rather than the bioavailability of the feedstock. Ebner *et al*.^8^ measured hydrolysis rates using bio-methane potential assays, and found that co-digestion increased hydrolysis rates when food waste and manure was co-digested compared to mono-digestion in BMP assays. The synergistic effect was attributed to dilution of inhibitory compounds and improved nutrient balance due to co-digestion^8,9^. The enzymes involved in hydrogenotrophic methanogenesis and syntrophic acetate oxidation requires trace elements such as selenium (Se), molybdenum (Mo) tungsten (W), cobolt (Co), nickel (Ni) and iron (Fe)^4^. Lack of these trace elements can limit the syntrophic acetate oxidation as well as formate oxidation^3–5^. The resulting accumulation of formate may again inhibit propionic acid oxidation. This will result in an overall acid accumulation, which eventually can cause the pH in the digester to drop, severely affecting or completely stopping the methanogenesis. Notably, the toxicity of intermediate compounds also increases with increasing temperature^10^ and thermophilic digesters are commonly considered to be more prone to process inhibition than mesophilic digesters^7^. Moreover, anaerobic digestion processes operating at high-temperature often selects for a less diverse microbial community, which is more vulnerable to stress and operational changes^11,12^. Most studies in the literature have focused on enhancing functionality and operation of anaerobic co-digesters using food waste and other feedstocks. Several studies have compared performance of mesophilic and thermophilic digesters^2,3^, but comparison of community structures and diversity in anaerobic digesters and anaerobic co-digesters at these different temperatures are rare in the literature.

Accordingly, the aim of this study was to investigate the microbial structure of co-digestion of food waste and cow manure under mesophilic (37 °C) and thermophilic (55 °C) conditions. Additionally, we compared the microbial structure of the co-digestion process to that of food waste digestion alone to determine how the co-digestion process influences the microbial communities. Also, performance parameters were studied under the various conditions and it was attempted to explain performance efficiencies using microbial data.

## Results and Discussion

### Performance of the biogas reactors

**Figure 1:**
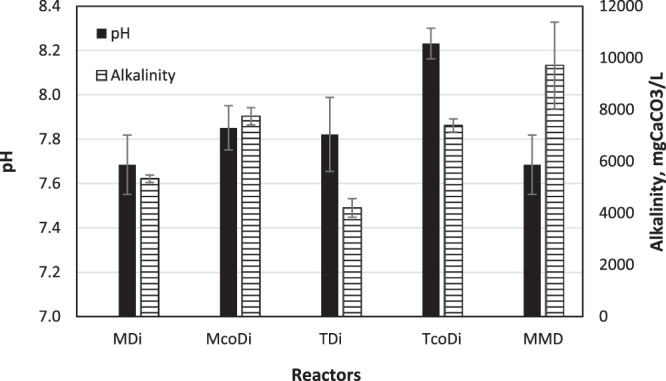
The pH and alkalinity in the various digesters, presented as an average of measurements over a period. For comparison, the values of pH and alkalinity in the mesophilic manure-only fed digester (MMD) is also presented.

**Figure 2:**
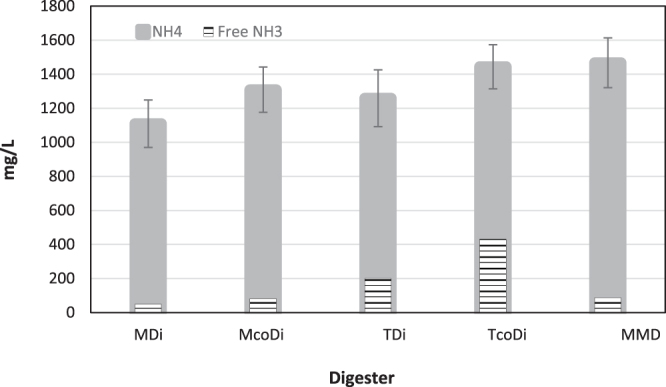
Total ammonia and free ammonia concentrations in the digestion and co-digestion systems.

The average pH values for MDi, McoDi, TDi and TcoDi were 7.7 ± 0.1, 7.9 ± 0.1, 7.8 ± 0.2 and 8.2 ± 0.1, respectively. As illustrated in Fig. 1, the average pH of McoDi and MDi was comparable, although slightly higher in McoDi. Notably, while the pH of TDi was similar to mesophilic processes, an elevated pH was clearly seen for TcoDi. This agrees with the higher ammonia concentrations in the co-digestion systems (McoDi and TcoDi), which were 16.5% and 13.7% higher than the MDi and TDi digesters, respectively (Fig. 2). Additionally, as shown in Fig. 1, alkalinity was also higher in the co-digesters than the digestion systems. This is most likely due to the addition of manure, as manure typically has high content of nitrogen-bearing material^8^ that are released as ammonia during the fermentation process and acts as a buffering system.

**Figure 3:**
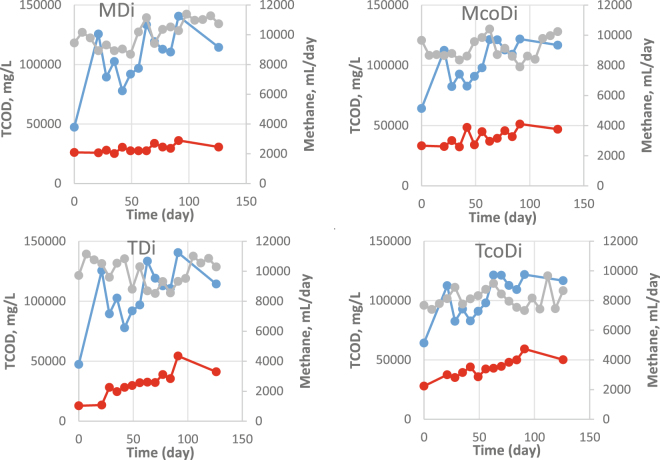
TCOD concentrations in the influent (blue) and effluent (red) and average methane generation (grey) from each digester.

The degradation of organic material in all the digesters was measured in terms of TCOD removal (Fig. 3). Regardless of the operational temperature, the removal efficiencies were higher for the digestion systems (MDi: 73.0% and TDi: 66.4%) than the co-digesters (McoDi: 61.4% and TcoDi: 56.7%). This was expected due to a general high degradability of food waste^13^. MDi had the highest methane yield of all four digesters with 479.5 ± 33.9 ml CH_4_/g VS_feed_, which was 11.5%, 7.0% and 31.6% higher than the McoDi, TDi and TcoDi, respectively (Fig. 3). These results are in agreement with earlier studies reported elsewhere^8,14^. Additionally, lower methane production in the thermophilic reactors may be related to the presence of higher free ammonia concentrations that was, on average, 198 mg/L for TDi and 431 mg/L in TcoDi (Fig. 2), potentially causing inhibition of the methanogenesis process^15^.

It has been observed that co-digestion of food waste and manure may enhance biogas production, and lead to more stable digestion processes^7,16–18^. We also observed higher methane production when we compared the methane yield of McoDi fed with the mixture of food waste and manure with that of manure-only fed mesophilic (37 °C) digester. The methane yields of manure-fed digester and McoDi were 133 ± 18 and 430 ± 28 mL CH_4_/g VS_feed_, respectively. Based on the measured specific methane yield from MDi and the manure-only reactor, the expected methane yield for McoDi without any synergistic effects would be 341 mL CH_4_/g VS_feed_. However, our results showed that the observed methane yield of McoDi was 430 mL CH_4_/g VS_feed_, meaning that the co-digestion of food waste and manure (McoDi) resulted in 26% higher methane production than the sum of digestions of individual substrates.

The solubilization values estimated for MDi, McoDi, TDi and TcoDi were 56, 55, 63 and 48%, respectively (see Equation 1). The highest solubilization extent was observed in the TDi (63%), although less methane was generated in this system as compared to MDi and McoDi (discussed above). It was also noticed that the SCOD fraction of the extent of solubilization in TDi was quite high (10%) and thus less solubilized compounds were converted into the final product methane. This accumulation of soluble COD was likely prompted by the higher degradability of the food waste as compared to manure. The SCOD accounted for 1, 2 and 6% of the solubilization extent in MDi, McoDi and TcoDi, respectively. Moreover, the TcoDi showed the lowest solubilization extent (48%), indicating less efficient solubilization of the substrate mixture. Additionally, when the SCOD results were compared between the digester sets (i.e., MDi vs. TDi and McoDi vs. TcoDi), it revealed that the SCOD was 10 and 4 times higher in the effluent of the TDi and TcoDi than those of MDi and McoDi. However, the effluent SCOD concentrations were statistically comparable in the mesophilic digester (932 ± 151) and co-digester (1537 ± 511) (p_value_ = 0.05).

**Figure 4:**
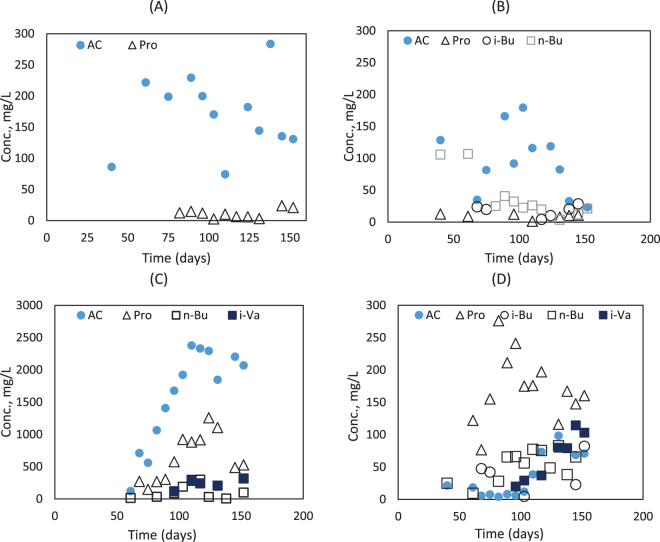
Volatile fatty acids concentrations in (**A**): MDi; (**B**) McoDi; (**C**): TDi; and (**D**): TcoDi digestion systems.

Overall, both mesophilic digesters had low concentrations of volatile fatty acids (VFAs). Analysis of the VFAs measurements (Fig. 4) revealed acetate as the main VFA in the mesophilic digesters MDi and McoDi, which was, on average, 172 ± 61 and 93 ± 54 mg/L, respectively. The remaining VFAs detected in the mesophilic digesters, propionic, iso-butyric and butyric acids, were all below 50 mg/L (Fig. 4). Thus, it appeared that the fermenting and methanogenic processes were in balance preventing accumulation of intermediate products in MDi and McoDi. Low VFAs concentrations were reported for an anaerobic digester operated under mesophilic condition for food waste treatment^19^. The analysis of the VFA profiles (Fig. 4) revealed a totally different behavior in the thermophilic digester TDi and co-digester TcoDi. Acetate and propionate accumulated in TDi, averaging 2150 ± 208 and 833 ± 282 mg/L, respectively. These results were consistent with previous works reported in literature that under thermophilic condition increased concentrations of VFAs were observed, while mesophilic digesters were capable of achieving lower VFA concentrations^19,20^. As can be seen from Fig. 4, the concentrations of total VFAs in TcoDi were less than 300 mg/L, in which propionate was the main VFA with an average concentration of 163 ± 27 mg/L. This difference in the VFA profile in TDi and TcoDi might be due to an improved synergistic performance of acetogens and methanogens in TcoDi that prevented the accumulation of the intermediate products and resulted in significantly lower concentrations of VFAs in TcoDi. The slow degradation of the manure which constituted 40% (on VS basis) of the feedstock in the co-digesters may also explain this difference.

### Microbial composition of the mesophilic reactors

**Figure 5:**
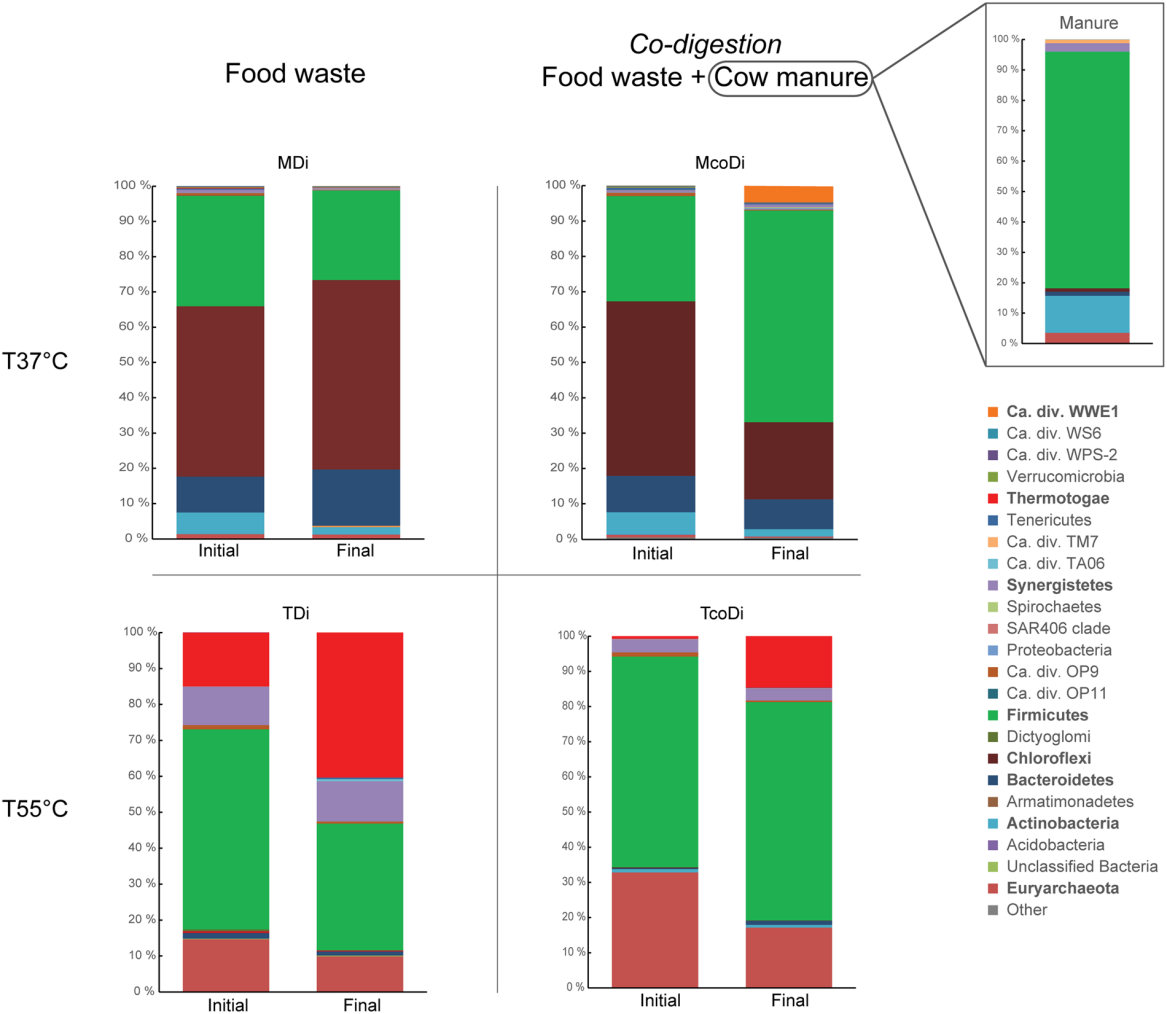
Phylogenetic distribution of the 16S rRNA gene sequences in anaerobic digesters and co-digesters, presented at phylum level. The effect of co-digestion was tested at mesophilic (T37 °C) and thermophilic (T55 °C) temperature.

Statistical analysis demonstrated that the anaerobic co-digestion process resulted in a significantly (p_value_ < 0.005) higher microbial richness compared to the digesters fed with food waste alone (see Supplementary Fig. S1). The major bacteria in both mesophilic digesters included *Firmicutes*, *Chloroflexi*, *Bacteroidetes* and *Actinobacteria* (Fig. 4). However, the distribution of these major bacteria in the digesters was different. *Chloroflexi*, which in the final phase constituted 54% of the sequences, was the dominant phylum in MDi, followed by 25% *Firmicutes* and 15% *Bacteroidetes*. *Firmicutes* (60% of the sequences in the final phase) was the dominant phylum in McoDi, while the relative abundance of *Chloroflexi* (22%) and *Bacteroidetes* (8%) was noticeably lower in McoDi than MDi. Additionally, the candidate phylum WWE1 was identified in McoDi and accounted for 5% of the relative abundance. Limam *et al*.^21^ investigated the metabolic function of WWE1 members and suggested that the members of this division were involved in hydrolysis of cellulosic materials. WWE1 was also found in mesophilic co-digestion studies of manure with various agricultural residues^22,23^. Thus, the addition of cow manure to the co-digestion system seems to spur the growth of WWE1 members, probably involved in decomposition of cellulose content of the manure. It should be noted that WWE1 was not detected in the cow manure in the current study. The dominance of *Chloroflexi* in MDi (Fig. 5), which was mainly made up of the T78 group of family *Anaerolinacea*, was probably due to the presence of fermentable carbohydrates in the preprocessed food waste used (pasteurized at 70 °C). *Anaerolinacea* are mostly saccharolytic anaerobes and use a number of carbohydrates for growth^24,25^. Use of manure in the feedstock of the co-digestion systems resulted in a different relative abundance of bacterial communities in McoDi and prompted the prevalence of *Firmicutes*, which include members with very versatile metabolic characteristics and more potential to degrade the recalcitrant manure^26,27^. *Firmicutes* has been reported as one of the major microbial contributors in several studies carried out on anaerobic digesters, indicating that the phylum is common in both mesophilic and thermophilic processes^28,29^. Additionally, *Firmicutes* dominance has also been linked to better reactor performance^20^. The higher relative abundance of *Bacteroidetes* in MDi, which was fed with the preprocessed food waste, probably indicates involvement of their members in degradation of intermediate degradation products of carbohydrates and proteins.

**Figure 6:**
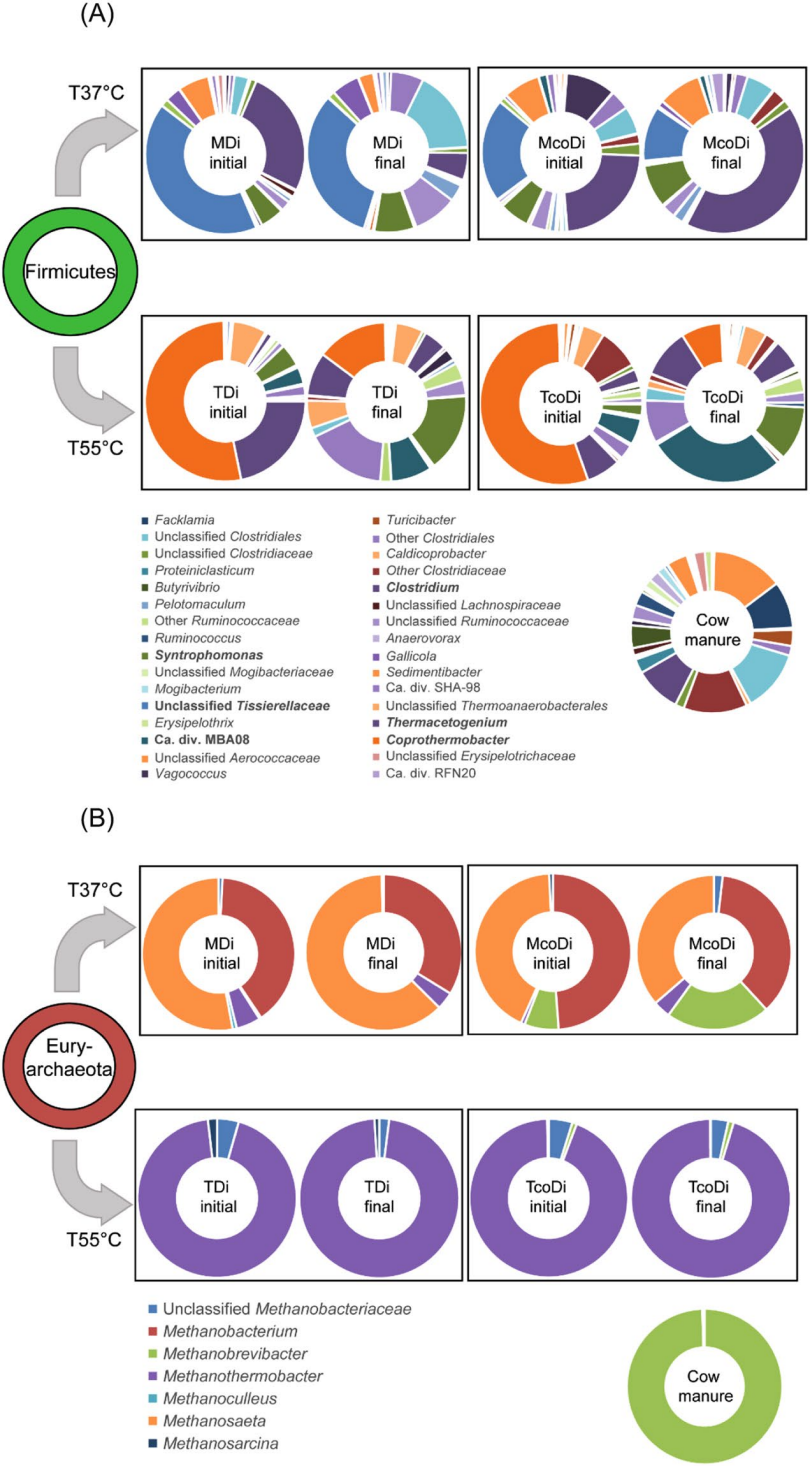
Phylogenetic distribution of the genera within (**A**) phylum *Firmicutes* and (**B**) phylum *Euryarchaeota*. Although all genera (>0.005% of total sequences) are included in the pie chart for *Firmicutes*, only the genera representing ≥1% of the sequences in at least one of the samples is included in the legend to reduce size. The most dominant genera are highlighted in bold type to ease the visual interpretation.

**Figure 7:**
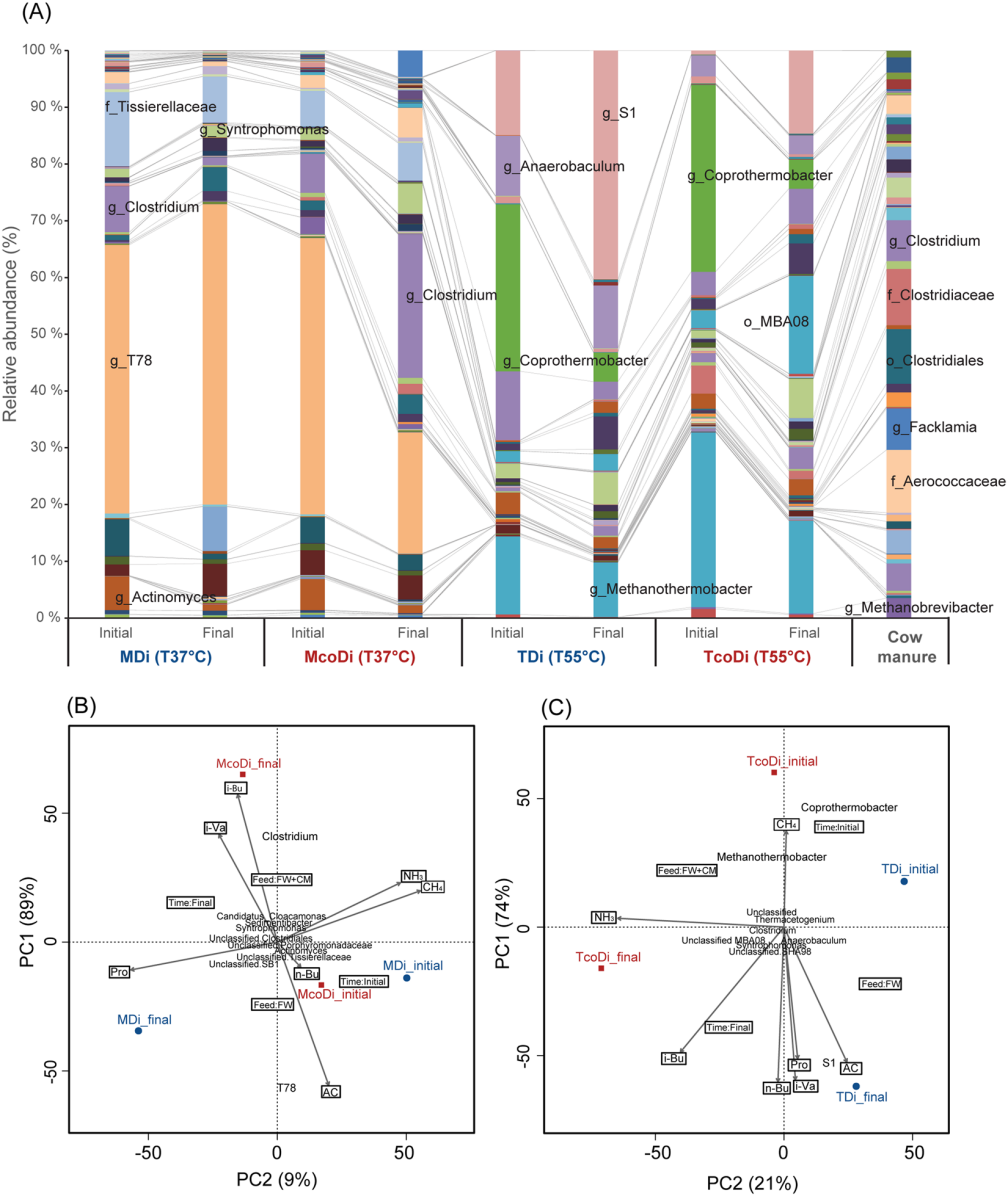
Relative abundance of the OTUs, classified at genus level or highest possible ranked taxonomic level (**A**), and their association with operational conditions and process variables in the mesophilic (**B**) and the thermophilic (**C**) digesters assessed through principal component analysis (PCA). The chemical variables included in the PCA plots are the values of NH_3_, CH_4_ (ml/week), and VFAs (propionate; “Pro”, Acetate; “AC”, n-Butyrate; “n-Bu”, i-Butyrate; “i-Bu”, i-Valerate; “i-Va”). Only the most abundant taxa are annotated in the barchart to reduce the complexity. A comprehensive OTU table is supplied in the Electronical supplementary material, Table S1.

Notably, the relative abundance of *Fimicutes* increased in the final phase of McoDi compared to MDi. This could be due to the addition of manure which is a potential source of *Firmicutes*, as organisms belonging to this phylum dominated the microbial profile of the manure feedstock with 78% of all sequences (Fig. 5). To evaluate this, the genus level distribution of the sequences was investigated and a high diversity within the *Firmicutes*-phylum was noticed (Fig. 6A). An unclassified genus of the family *Tissierellaceae* accounted for 32% of the sequences assigned to the phylum *Firmicutes* in the final phase of MDi, while this value was much lower in McoDi (11% of phylum) where the main genus was *Clostridium* (42% of phylum). In compliance, three OTUs assigned to *Clostridium* were significantly more abundant in McoDi compared to MDi (Supplementary Fig. S2, see also Fig. 7A). Thus, it would appear that *Firmicutes* in general and *Clostridium* in particular played an important role in McoDi system. This genus was also represented in the cow manure samples, accounting for 9% of the *Firmicutes*-related sequences. A principle component analysis (PCA) was used to investigate possible links between microbiome and performance. Based on this analysis an association of *Clostridium* to the concentration of n-Butyrate was observed, although only low levels of butyrate were measured in both mesophilic digesters (Fig. 7B). Notably, a correlation was observed between the abundance of *Clostridium* and the cow manure used in the feedstock mixture of the co-digestion system. It is therefore reasonable to believe that the increase in relative abundance of *Clostridium* in the co-digestion system was originated from the cow manure as a feedstock. It should nevertheless be mentioned that some *Clostridium* species can form endospores that enable them to tolerate moist heat^30^ and pasteurization pretreatment applied on the food waste collected from the processing center. The food waste can therefore not be eliminated as a source of *Clostridium*. However, a carry-over from the cow manure used seems more likely due to the abovementioned increase of *Clostridium* in McoDi. This was further supported by the correlation of higher numbers of *Clostridium* with the addition of cow manure.

Archaea belonging to the phylum *Euryarchaeota* were dominated by *Methanobacterium* and *Methanosaeta* in both MDi and McoDi (Fig. 6B). The genus distribution within this phylum was similar for initial and final phase in MDi, where *Methanosaeta* was dominant, constituting 53% and 62% (in initial and final phase, respectively) of the archaeal sequences. *Methanosaeta* was also prominent in the McoDi (43% and 36% in initial and final phase, respectively), yet significantly lower compared to MDi (Supplementary Fig. S2). In addition to *Methanobacterium* and *Methanosaeta*, a noticeable portion of the methanogenic population was also assigned to the genus *Methanobrevibacter* in McoDi, with increasing relative abundance over time (from 7% of the archaeal sequences in the initial phase, to 22% in the final phase). *Methanobrevibacter* most likely originated from the manure used in this study as the analysis of manure samples showed *Methanobrevibacter* as the only dominant archaea (Fig. 6B). While *Methanosaeta* is known as an acetate-utilizing methanogen, *Methanobacterium* and *Methanobrevibacter* both contain H_2_ utilizing methanogens^31^, suggesting a mixed pathway for methane production in the mesophilic co-digestion system. The reason for the presence of high hydrogen utilizers might partly be due to slightly higher free ammonia observed in McoDi (81 mg/L vs. 50 mg/L in MDi) and partly due to the continual addition of *Methanobrevibacter* through manure. This agrees with previous studies indicating the dominance of hydrogen utilizing methanogens in manure-fed digesters^32,33^.

### Microbial composition in the thermophilic digesters

The bacterial communities in TDi and TcoDi were mainly composed of the four phyla (relative abundancy >1%) *Thermotogae*, *Firmicutes*, *Synergistetes* and *Bacteroidetes* (Fig. 5). However, the relative abundance of these phyla was remarkably different within TDi and TcoDi, except for *Bacteroidetes* that accounted for 1–2% of the total sequences in both digesters. The profound effect of co-digestion on the bacterial community in the final phase of TcoDi was reflected by increased relative abundance of *Firmicutes* (62% of all sequences) and a decreased relative abundance of *Thermotogae* (15% of all sequences). In comparison, the relative abundance of *Firmicutes* and *Thermotogae* was 35% and 40% in the TDi, respectively. Notably, the distribution of *Synergistetes*, mainly represented by genus *Anaerobaculum*, differed significantly in the thermophilic digesters (Supplementary Fig. S2), accounting 11% and 4% in the final phase of TDi and TcoDi, respectively. The members of this taxon fermentatively convert polypeptides and organic acids to acetate, H_2_ and CO_2_^34^. Compared to the initial phase, the relative abundance of *Thermotogae* increased over time in both thermophilic reactors, while the relative abundance of *Firmicutes* decreased only in TDi (Fig. 5). Similar to the mesophilic digesters, co-digestion of manure and food waste seemingly spurred the growth and dominance of *Firmicutes* in the TcoDi, while the digestion of food waste alone induced more even-distribution of *Thermotoga* and *Firmicutes* and supported the development of more *Synergistetes* in TDi.

While phylotypes assigned to genus *Coprothermobacter* accounted for more than 50% of the sequences assigned to *Firmicutes* in the initial samples of both TDi and TcoDi, this portion was largely reduced in the final phase (15% and 8% of *Firmicutes*, in TDi and TcoDi respectively). Instead, a more even distribution of several genera was observed in the final phase (Fig. 6A), with prominence of *Syntrophomonas* (16% and 11% of *Firmicutes*, in TDi and TcoDi respectively), *Thermactogenium* (9% and 10% in TDi and TcoDi respectively) and unclassified phylotypes assigned to the candidate divisions SHA-98 (17% and 9% in TDi and TcoDi respectively) and MBA08 (8% and 28% in TDi and TcoDi respectively). *Coprothermobacter* is a proteolytic bacterium involved in the syntrophic fermentation of polypeptides, and the high dominance in the initial phase was most likely reflected by a strong dominance of the *Coprothermobacter* population in the seed culture from the FREVAR biogas plant, as reported in a previous study^35^. The OTUs assigned to genus *Thermacetogenium* were in all probability affiliated to *Thermacetogenium phaeum* ^35^, a bacterium able to oxidize acetate syntrophically and grow acetogenically on organic acids and alcohols^36,37^.

The phylotype affiliated to candidate order MBA08 was noticeably higher in the TcoDi compared to TDi. This probably suggests the role of the members of the candidate group MBA08 in the co-digestion of food waste and manure. Li^38^ also reported the candidate order MBA08 as one of the major bacterial groups in the thermophilic reactor of a staged system used for the co-digestion of whey and manure. However, none of the *Firmicutes*-associated sequences obtained from cow manure were related to MBA08 in the current study. On the contrary, the relative abundance of the candidate order SHA-98 was greater in the food waste-fed digester (i.e., TDi). There is almost no knowledge regarding the function of the members of the unclassified order SHA-98 and no genera could be assigned in this group.

Most of the phylotypes that could be assigned to a known genus in the order *Clostridiales* were probably involved in the degradation of polysaccharides, fermentable carbohydrates and syntrophic oxidation of saturated fatty acids^26^. Members of *Syntrophomonas* are believed to oxidize anaerobically C_4_-C_18_ saturated fatty acids^39^. *Clostridium* consists of bacteria that display metabolic versatility^26,40^. *Ruminococccaceae*, *Caldicoprobacteriaceae* and *Lachnospiraceae* were less dominant families. *Caldicoprobacter*, which exclusively was represented by *Caldicoprobacteriaceae* in both digesters, ferments xylan and simple sugars to lactate, acetate, H_2_ and CO_2_^41^. Interestingly, the family *Lachnospiracea*, although less abundant, differed in the genus and was mainly composed of *Butyrivibrio* in TcoDi and of *Coprococcus* in TDi. Members of the *Butyrivibrio* and *Coprococcus* both use fermentable carbohydrates, however, the *Butyrivibrio* members are also involved in degradation of plant materials and are a major component of rumen microbiota^41^. The *Butyrivibrio* members probably came from cow manure and were able to retain their activities and growth in TcoDi.

Unlike the large diversity observed within *Firmicutes*, the second most dominant phylum, *Thermotogae* (Fig. 5), demonstrated very low diversity as almost all sequences were assigned to the candidate division *Thermotoga* S1. Notably, a clear difference was found in the relative abundance of this phylum in the final phase of TDi (40% of all sequences) and TcoDi (15% of all sequences). As described earlier^42^, the members of *Thermotoga* are capable to grow on the various simple (e.g., glucose) and complex (e.g., xylan and starch) polysaccharides. The lower abundance of *Thermotoga* species may explain the lower methane yield in the TcoDi (23% less) than the TDi, especially considering the higher amount of particulate COD (44% higher) that left the TcoDi as compared to that of the TDi. Presence of the higher particulate COD might be due to an inefficient conversion of the complex carbohydrates in the feed, in particular in the recalcitrant manure. Additionally, principle component analysis (Fig. 7C) showed a correlation between the relative abundance of S1 and the concentrations of VFAs, indicating that the elevated concentrations of VFAs in TDi could be a cause-effect of an enhanced degradation of polysaccharides by *Thermotoga* S1. Furthermore, the free ammonia measured was 2.2 times greater in TcoDi (431 mg/L) than TDi (198 mg/L), suggesting that ammonium may possibly influence the abundance of the *Thermotoga* species^43^.

Overall, it would appear that the detection of higher relative abundance of 16S rRNA genes assigned to the genera *Anaerobaculum*, *Coprothermobacter*, *Thermotoga* and *Syntrophomonas* in TDi might indirectly imply an enhanced hydrolysis and acidogenesis of the food waste as compared to the co-digestion of food waste and manure. This might further be supported by the presence of significantly greater amount of VFAs (acetate, propionate and butyrate) in TDi than TcoDi (Fig. 4).

Analysis of the archaeal sequences (Fig. 6) showed that the process configuration (digestion vs. co-digestion) had little influence on the composition of methanogens and that the genus *Methanothermobacter*, which contains hydrogen utilizers, was almost entirely predominant in TDi and TcoDi. A correlation between *Methanothermobacter* and *Coprothermobacter* was observed, as well as an association with methane production (Fig. 7C). Such co-existence has frequently been reported in literature, drawing a scenario of a synergic relationship where *Coprothermobacter* supply *Methanothermobacter* with hydrogen^44^. The dominance of *Methanothermobacter* agrees with a previous study on community structure in a thermophilic biogas plant (FREVAR), from where the inoculum was taken for the start-up of the thermophilic digesters used in this study^35^. The lack of *Methanosaetaceae* species reflected an unfavorable environment (e.g., high free ammonia content) for their activities in TDi and TcoDi, suggesting the prevalence of the hydrogenotrophic methanogensis pathway in both digesters. In addition to an unfavorable environment, the prevalence of *Methanothermobacter* members might be due to the improved hydrolysis and fermentation at the elevated temperature that required syntrophic reactions to efficiently convert intermediates such as H_2_ and carboxylic organic acids.

## Conclusion

The anaerobic digesters fed solely food waste performed better than the co-digesters (food waste and cow manure), most probably due to the addition of a more recalcitrant material in the form of cow manure in the co-digesters. Nevertheless, co-digestion resulted in a higher microbial diversity at both temperatures, compared to anaerobic digestion of food waste as sole substrate. This could be a reflection of the increased complexity of feedstocks in co-digestion, selecting for a richer microbial community. Although similar in the initial phase, the microbial community compositions diverged when cow manure was added at both temperatures. Based on our observations, we speculate that this variation is mostly explained by cow manure providing trace minerals and a balanced C/N ratio, rather than carry-over of microorganisms from the cow manure. However, the increased population *Clostridium* in both McoDi and TcoDi indicates that the establishment of this population is a direct result of microbiome transmission from the cow manure. Carry-over of methanogens from the cow manure, represented by *Methanobrevibacter* was also suggested for the mesophilic co-digestion system (McoDi), while only to a minor extent in the thermophilic co-digestion system (TcoDi). As higher microbial diversity often is associated with a microbiome that is more resilient to environmental changes and stress, co-digestion could potentially enhance the robustness of the anaerobic digestion process. Additionally, co-digestion at mesophilic temperature clearly showed a synergistic effect, yielded more methane than the digestion of manure-alone.

## Materials and Methods

### Feedstock

Food waste (FW) was shipped from Norsk Matretur AS (Norwegian food recycling, Finstadjordet, Norway), which is a central food waste pre-treatment plant which reduces particle size to <7 mm and sanitizes the waste at 70 °C for 1 hour, as required by Norwegian health regulations. Dairy cow manure was collected at the farm of the Norwegian University of Life Sciences (Ås, Norway). Both manure and FW were stored at 4 °C and diluted using tap water to achieve the targeted organic loading rate. The waste batch were characterized on a weekly basis to ensure a constant organic loading. The average characteristics of the substrates are shown in Table 1.

**Table 1:** Food waste and manure characteristics used in the experiments (average ± standard deviation).

Parameters	Unit	Food waste	Manure
**Total Solids**	%	17.8 ± 1.2	7.2 ± 0.6
**Volatile Solids**	%	16.1 ± 1.2	5.7 ± 0.6
**VS/TS**		0.90	0.8
**TCOD**	g/L	271 ± 57.5	90.0 ± 17.5
**TCOD/VS**		1.7 ± 0.3	1.6 ± 0.3
**SCOD**	g/L	95 ± 12	16.0 ± 7.0
TAN*	mg/L	504 ± 153	1324 ± 152
**pH**		3.9 ± 0.1	7.5 ± 0.1
**Acetate**	mg/L	44642 ± 16576	2159 ± 1241
**Propionate**	mg/L	1251 ± 547	736 ± 182
**i-Butyrate**	mg/L	212 ± 14	75 ± 48
**n-Butyrate**	mg/L	244 ± 57	49 ± 44

^*^Total ammonia nitrogen.

### Set up and operation of digesters

Four completely mixed reactors (Belach Bioteknik, Sweden) with a working volume of 6 L were used in this study. Two of the digesters were only fed with food waste, where one of them was kept at mesophilic temperature (37 ± 0.1 °C; MDi), while the other one was kept at thermophilic temperature (55 ± 0.1 °C; TDi). The Co-digestion systems consisted of one mesophilic co-digester (McoDi) and one thermophilic co-digester (TcoDi), which both were fed with a mixture of food waste and cow manure in a ratio of 60:40 on volatile solid (VS) basis. The start-up of the reactors was performed as previously described by Estevez *et al*.^45^. Seed sludge for the mesophilic reactors came from the Oslo EGE biogas plant (Nes, Norway); a full scale mesophilic anaerobic digester with food waste as its sole substrate. Seed sludge for the thermophilic reactors came from the FREVAR biogas plant (Fredrikstad, Norway); a full scale thermophilic reactor with sludge and food waste as its substrates. The feeding of the experimental reactors was done manually once per day, 6 days a week. Hydraulic retention time (HRT) and organic loading rate (OLR) for all the digesters were 20 days and 3 g VS/L/d, respectively. Temperature, pH, biogas volume and stirrer speed (set at 100 rpm) of the digesters were monitored and recorded online using BIOPHANTOM software (Belach Bioteknik, Sweden). Additionally, samples of the effluent were regularly taken to monitor the performance of the digesters and co-digesters.

### Analytical methods

Total solids (TS), volatile solids (VS), total chemical oxygen demand (TCOD) and soluble chemical oxygen demand (SCOD) were measured following Standard Methods^46^. Chemical oxygen demand was measured using Merck Spectroquant® COD Cell test with measuring range 0.5–10 g/L. The extent of solubilization was calculated using Equation (1).

1 $$Extent\,of\,solubilization\,( \% )=\frac{CO{D}_{CH4}+SCOD-SCO{D}_{in}}{PCO{D}_{in}}$$

where COD_CH4_ is the COD equivalent of the CH_4_ produced; SCOD is effluent soluble COD; SCOD_in_ is influent soluble COD and PCOD_in_ is the influent particulate COD.

Ammonium (NH_4_^+^) was measured with an ammonium ion selective electrode according to the company’s manual (Orion 93; Thermoscientific, USA). Biogas composition was analyzed online for methane and carbon dioxide as previously described Zamanzadeh *et al*.^15^, with the use of an SRI gas chromatograph (Model 8610 C) equipped with a thermal conductivity detector (TCD, RCH3100, USA) and a 2 m Haysep-D column. Volatile fatty acids (VFAs) were determined by high-pressure liquid chromatography (HPLC; Dionex 3100, USA) with a Zorbax Eclipse Plus C18 column (150 × 2.1 mm column; 3.5 µm particles; Agilent, USA) as previously described by Zamanzadeh *et al*.^15^. The samples were centrifuged at 14000 rpm for 5 min, adjusted to pH < 2.5 using 95–98% H_2_SO_4_ and then filtered through a 0.45 µm cellulose acetate syringe filter.

### DNA extraction

Samples were collected for 16S rRNA gene sequencing during the initial stable phase of the anaerobic digestion (after 68 days) and in the final phase (after 152 days) from reactors MDi, McoDi, TDi and TcoDi, in addition to the cow manure used for co-digestion. Food waste was also sampled for the same purpose, but genomic DNA was not successfully recovered, likely due to the sanitization treatment (70 °C for 1 hour). All samples were frozen immediately after sampling, and stored at −20 °C until DNA extraction. For DNA extraction, thawed samples were centrifuged at 18 800 x g for 7 min. to remove the liquid. The pellet was then re-suspended in S.T.A.R buffer (Roche Diagnostics Corporation, USA) to stabilize nucleic acid and prevent bacterial growth. The suspension was vortexed followed by a subsequently slow spin to dissociate cells from large particles. The cell-containing suspension was transferred to FastPrep24 tubes with acidic washed glass beads for mechanical lysis. DNA was extracted using a commercial DNA extraction kit (LGC Genomics, UK), and DNA concentration measured with Qubit™ fluorometer and Quant-iT™ dsDNA BR Assay Kit (Invitrogen, USA). The DNA samples were stored at −20 °C until sequencing preparation.

### 16 S rRNA gene sequencing

The 16S rRNA gene amplicons were prepared for the Illumina MiSeq system (Illumina Inc.) as described in Zamanzadeh *et al*.^15^. In brief, 16S rRNA gene PCR amplification was carried out using the Pro341F/Pro805R primer set selected from Takahsahi *et al*.^47^ modified with an Illumina adapter overhang in, and iProof HF DNA polymerase (BioRad, USA). A second PCR was carried out to attach unique 8-bp indices (Nextera XT Index Kit) to the Illumina sequencing adaptors to allow multiplexing of samples. A clean-up step (Agencourt AMPure XP beads, Beckman Coulter, USA) was preformed after each PCR. The amplicons were quantified (Quant-iT™ dsDNA HS Assay Kit and Qubit™ fluorometer, Invitrogen, USA), normalized and pooled to equimolar concentration, and then spiked with 30% PhiX control. A final concentration of 8 pm denaturated DNA was sequenced on an Illumina MiSeq instrument using the MiSeq reagent kit V3.

### Sequencing analysis

All 16S rRNA gene sequences were processed using the QIIME version 1.8.0 software package^48^. Single-ends were trimmed to 200 bp and quality filtered as follows: only three sequential low-quality (Phred quality score <20) bases were allowed per sequence before truncating, and sequences with <75% (of total length) consecutive high-quality base calls were discarded. No N characters or barcodes were allowed in the sequence. Chimeric sequences were removed from the dataset using UCHIME incorporated in USEARH^49^ and a threshold of 3% dissimilarity between 16S rRNA gene sequences was used to cluster sequences into *de novo* operational taxonomic units (OTUs)^49^. Taxonomy (up to rank ‘genus’) was assigned to each OTU using the uclust-based consensus taxonomy assigner implemented in QIIME with default parameters. Alpha rarefaction plot of the phylogenetic diversity was generated using the script alpha_rarefaction.py with default parameters in QIIME. Low abundant OTUs (those with a total count less than 0.005%) and singletons were filtered out. Statistical analysis and visualization was carried out using Calypso version 8.20^50,51^. The alpha diversity of the microbial communities was measured by OTU richness in addition to the Shannon index. The diversity were further compared with ANOVA to evaluate the significance between the different subgroups (digestion vs. co-digestion, mesophilic vs. thermophilic). ANOVA was also used to compare taxa abundance across the different digesters. Finally, associations between reactor performance and the community composition were assessed. For this, a principle component analysis (PCA) was applied to examine how much of the variance in the 16S rRNA gene sequencing dataset could explain the process variables (day-specific concentration of VFA and NH_3_, and weekly measurements of CH_4_ production).

### Data Availability

Sequence data are available at NCBI Sequence Read Archive under accession number SRP123045.

## Electronic supplementary material

Figures S1 and S2

Table S1

## References

[1] Aichinger P (2015). Synergistic co-digestion of solid-organic-waste and municipal-sewage-sludge: 1 plus 1 equals more than 2 in terms of biogas production and solids reduction. Water Res..

[2] Marañón E (2012). Co-digestion of cattle manure with food waste and sludge to increase biogas production. Waste Manag..

[3] Zhang L, Lee YW, Jahng D (2011). Anaerobic co-digestion of food waste and piggery wastewater: Focusing on the role of trace elements. Bioresour. Technol..

[4] Banks CJ, Zhang Y, Jiang Y, Heaven S (2012). Trace element requirements for stable food waste digestion at elevated ammonia concentrations. Bioresour. Technol..

[5] Zhang W, Zhang L, Li A (2015). Enhanced anaerobic digestion of food waste by trace metal elements supplementation and reduced metals dosage by green chelating agent [S, S]-EDDS via improving metals bioavailability. Water Res..

[6] Edelmann W, Engeli H, Gradenecker M (2000). Co-digestion of organic solid waste and sludge from sewage treatment. Water Science and Technology.

[7] Labatut RA, Angenent LT, Scott NR (2014). Conventional mesophilic vs. thermophilic anaerobic digestion: a trade-off between performance and stability?. Water research.

[8] Ebner JH, Labatut RA, Lodge JS, Williamson AA, Trabold TA (2016). Anaerobic co-digestion of commercial food waste and dairy manure: Characterizing biochemical parameters and synergistic effects. Waste Management.

[9] Astals S, Batstone D, Mata-Alvarez J, Jensen P (2014). Identification of synergistic impacts during anaerobic co-digestion of organic wastes. Bioresource technology.

[10] Angelidaki I, Ahring BK (1993). Thermophilic anaerobic digestion of livestock waste: the effect of ammonia. Applied Microbiology and Biotechnology.

[11] Karakashev D, Batstone DJ, Angelidaki I (2005). Influence of environmental conditions on methanogenic compositions in anaerobic biogas reactors. Applied and Environmental Microbiology.

[12] Levén L, Eriksson ARB, Schnürer A (2007). Effect of process temperature on bacterial and archaeal communities in two methanogenic bioreactors treating organic household waste. FEMS microbiology ecology.

[13] Fisgativa H, Tremier A, Dabert P (2016). Characterizing the variability of food waste quality: a need for efficient valorisation through anaerobic digestion. Waste Management.

[14] El-Mashad HM, Zhang R (2010). Biogas production from co-digestion of dairy manure and food waste. Bioresource technology.

[15] Zamanzadeh M, Hagen LH, Svensson K, Linjordet R, Horn SJ (2016). Anaerobic digestion of food waste-effect of recirculation and temperature on performance and microbiology. Water Research.

[16] Zhang C, Xiao G, Peng L, Su H, Tan T (2013). The anaerobic co-digestion of food waste and cattle manure. Bioresource technology.

[17] Agyeman FO, Tao W (2014). Anaerobic co-digestion of food waste and dairy manure: Effects of food waste particle size and organic loading rate. Journal of environmental management.

[18] Pagés-Díaz J, Pereda-Reyes I, Taherzadeh MJ, Sárvári-Horváth I, Lundin M (2014). Anaerobic co-digestion of solid slaughterhouse wastes with agro-residues: synergistic and antagonistic interactions determined in batch digestion assays. Chemical Engineering Journal.

[19] Guo CL (2014). Effects of temperature and organic loading rate on the performance and microbial community of anaerobic co-digestion of waste activated sludge and food waste. Chemosphere.

[20] Wang, P., Wang, H., Qiu, Y., Ren, L., & Jiang, B. Microbial characteristics in anaerobic digestion process of food waste for methane production–A review. *Bioresource Technology*, In Press (2017). 10.1016/j.biortech.2017.06.152 28779951

[21] Limam RD (2014). Members of the uncultured bacterial candidate division WWE1 are implicated in anaerobic digestion of cellulose. MicrobiologyOpen.

[22] Sun L, Pope PB, Eijsink VG, Schnürer A (2015). Characterization of microbial community structure during continuous anaerobic digestion of straw and cow manure. Microbial biotechnology.

[23] Stolze Y (2016). Identification and genome reconstruction of abundant distinct taxa in microbiomes from one thermophilic and three mesophilic production-scale biogas plants. Biotechnology for Biofuels.

[24] Yamada T (2006). Anaerolineathermolimosa sp. nov., Levilineasaccharolytica gen. nov., sp. nov. and Leptolineatardivitalis gen. nov., sp. nov., novel filamentous anaerobes, and description of the new classes Anaerolineae classis nov.andCaldilineae classis nov.in the bacterial phylum Chloroflexi. Int. J. Syst. Evol. Microbiol..

[25] Sekiguchi Y (2003). Anaerolineathermophila gen. nov., sp. nov. and Caldilineaaerophila gen. nov., sp. nov., novel filamentous thermophiles that represent a previously uncultured lineage of the domain Bacteria at the subphylum level. Int. J. Syst. Evol. Microbiol..

[26] Riviere D (2009). Towards the definition of a core of microorganisms involved in anaerobic digestion of sludge. The ISME journal.

[27] Vanwonterghem I, Jensen PD, Ho DP, Batstone DJ, Tyson GW (2014). Linking microbial community structure, interactions and function in anaerobic digesters using new molecular techniques. Curr. Opin. Biotechnol..

[28] Sundberg C (2013). 454 pyrosequencing analyses of bacterial and archaeal richness in 21 full-scale biogas digesters. FEMS microbiology ecology.

[29] Guo X, Wang C, Sun F, Zhu W, Wu W (2014). A comparison of microbial characteristics between the thermophilic and mesophilic anaerobic digesters exposed to elevated food waste loadings. Bioresource technology.

[30] Dürre, P. Physiology and sporulation in Clostridium. In The Bacterial Spore: from Molecules to Systems (pp. 315–329). American Society of Microbiology (2016).

[31] Zinder, S. H. Physiological ecology of methanogens. In Methanogenesis (pp. 128–206). Springer US (1993).

[32] Demirel B, Scherer P (2008). The roles of acetotrophic and hydrogenotrophic methanogens during anaerobic conversion of biomass to methane: a review. Reviews in Environmental Science and Bio/Technology.

[33] Karakashev D, Batstone DJ, Trably E, Angelidaki I (2006). Acetate oxidation is the dominant methanogenic pathway from acetate in the absence of Methanosaetaceae. Applied and environmental microbiology.

[34] Menes RJ, Muxí L (2002). Anaerobaculum mobile sp. nov., a novel anaerobic, moderately thermophilic, peptide-fermenting bacterium that uses crotonate as an electron acceptor, and emended description of the genus Anaerobaculum. International journal of systematic and evolutionary microbiology.

[35] Hagen LH (2016). Quantitative metaproteomics highlight the metabolic contributions of uncultured phylotypes in a thermophilic anaerobic digester. Applied and Environmental Microbiology.

[36] Etchebehere C, Pavan ME, Zorzopulos J, Soubes M, Muxi L (1998). Coprothermobacter platensis sp. nov., a new anaerobic proteolytic thermophilic bacterium isolated from an anaerobic mesophilic sludge. International journal of systematic bacteriology.

[37] Hattori S, Kamagata Y, Hanada S, Shoun H (2000). Thermacetogenium phaeum gen. nov., sp. nov., a strictly anaerobic, thermophilic, syntrophic acetate-oxidizing bacterium. International Journal of Systematic and Evolutionary Microbiology.

[38] Li, Y. F. An integrated study on microbial community in anaerobic digestion systems (Doctoral dissertation, The Ohio State University) (2013).

[39] Zhao H, Yang D, Woese CR, Bryant MP (1993). Assignment of fatty acid-β-oxidizing syntrophic bacteria to Syntrophomonadaceae fam. nov.on the basis of 16S rRNA sequence analyses. International journal of systematic bacteriology.

[40] Hippe H, Andreesen J, Gottschalk G (1992). The genus Clostridium—nonmedical. The prokaryotes.

[41] Yokoyama H, Wagner ID, Wiegel J (2010). Caldicoprobacter oshimai gen. nov., sp. nov., an anaerobic, xylanolytic, extremely thermophilic bacterium isolated from sheep faeces, and proposal of Caldicoprobacteraceae fam. nov. International journal of systematic and evolutionary microbiology.

[42] De Vos, P. *et al*. Bergey’s Manual of Systematic Bacteriology Volume 3: The Firmicutes. 2nd Edition. Springer, New York (2009).

[43] Conners SB (2006). Microbial biochemistry, physiology, and biotechnology of hyperthermophilic Thermotoga species. FEMS microbiology reviews.

[44] Pervin HM (2013). Drivers of microbial community composition in mesophilic and thermophilic temperature-phased anaerobic digestion pre-treatment reactors. Water research.

[45] Sasaki K (2011). Syntrophic degradation of proteinaceous materials by the thermophilic strains Coprothermobacter proteolyticus and Methanothermobacter thermautotrophicus. Journal of Bioscience and Bioengineering.

[46] Estevez MM, Sapci Z, Linjordet R, Schnürer A, Morken J (2014). Semi-continuous anaerobic co-digestion of cow manure and steam-exploded Salix with recirculation of liquid digestate. J. Environ. Manage..

[47] APHA, AWWA & WEF. Standard Methods for the Examination of Water and Wastewater, twentiethed. American Public Health Association, Washington, DC., USA (1998).

[48] Takahashi S, Tomita J, Nishioka K, Hisada T, Nishijima M (2014). Development of a Prokaryotic Universal Primer for Simultaneous Analysis of Bacteria and Archaea Using Next-Generation Sequencing. PloS one.

[49] Caporaso JG (2010). QIIME allows analysis of high-throughput community sequencing data. Nature methods.

[50] Edgar RC, Haas BJ, Clemente JC, Quince C, Knight R (2011). UCHIME improves sensitivity and speed of chimera detection. Bioinformatics.

[51] Zakrzewski M (2016). Calypso: a user-friendly web-server for mining and visualizing microbiome–environment interactions. Bioinformatics.

