# Supplementary material for: Biogas production from food waste via co-digestion and digestion- effects on performance and microbial ecology
Source: Sci Rep. 2017 Dec 15;7:17664. doi: 10.1038/s41598-017-15784-w (PMC5732306; doi:10.1038/s41598-017-15784-w)
Supplement: Supplementary file 1 — Figures S1 and S2 [file 41598_2017_15784_MOESM1_ESM.pdf]

Supplementary Information:

Biogas production from food waste via co-digestion and digestion - effects on performance and microbial ecology

Mirzaman Zamanzadeh[a,b,c], Live H. Hagen[a], Kine Svensson[d], Roar Linjordet[d], Svein J. Horn[a*]

SUPPLEMENTARY FIGURES:

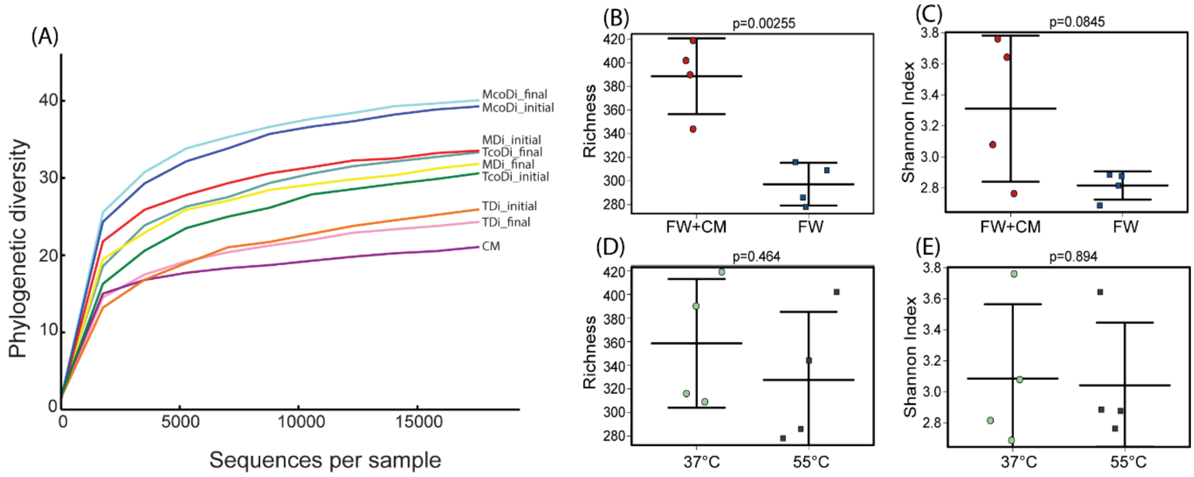

Figure S1: Diversity analysis. The alpha rarefraction curves (A) for all four reactors and cow manure (CM) was measured as phylogenetic diversity. Microbial diversity during co-digestion of food waste and cow manure (FW+CM), and digestion of only food waste (FW) was measured by richness (B) and Shannon index (C). Richness (D) and Shannon index (E) was also used to investigate the diversity in mesophilic (37°C) and thermophilic (55°C) processes. The p-value (Anova) is indicated above each stripchart.

(A)

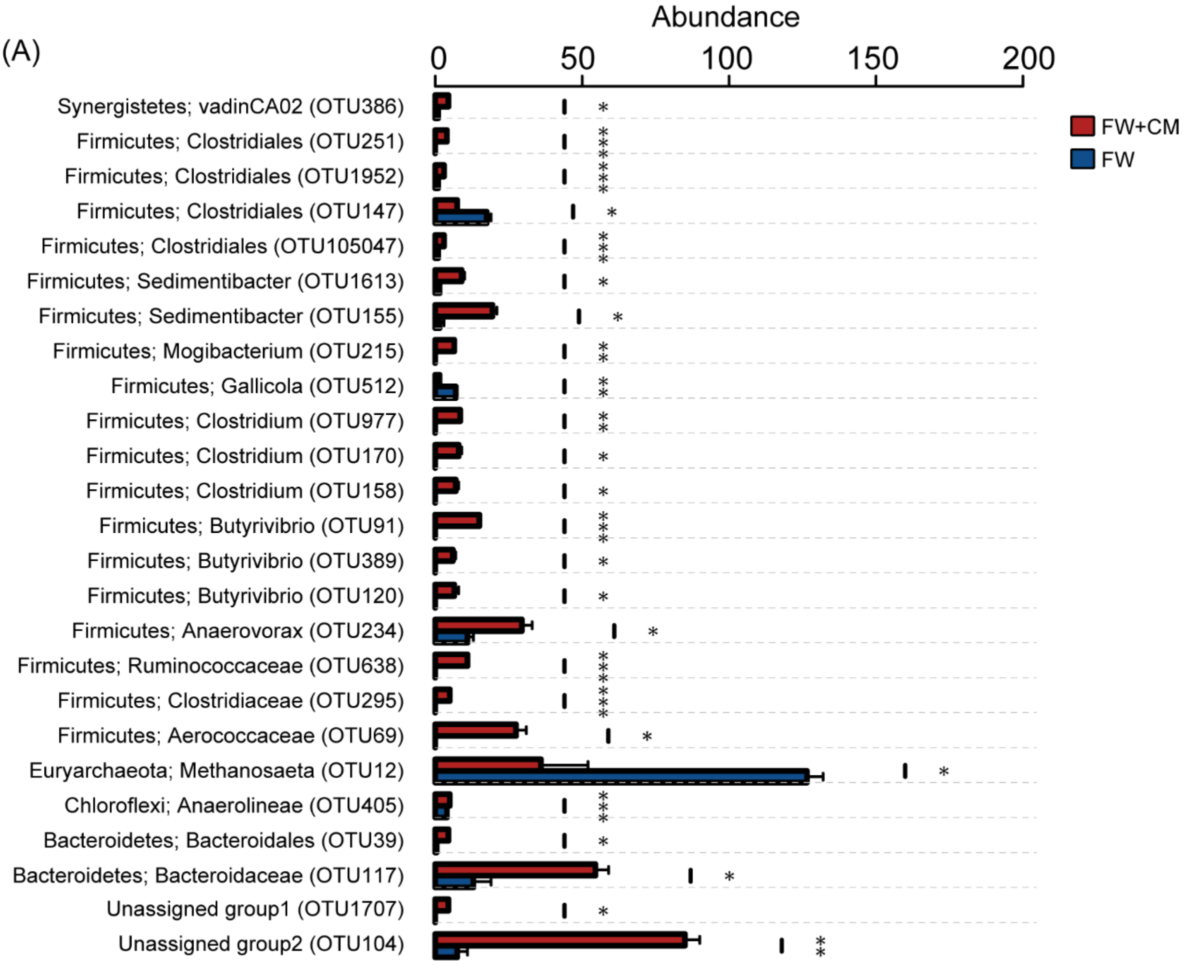

Figure continues on next page

Continued

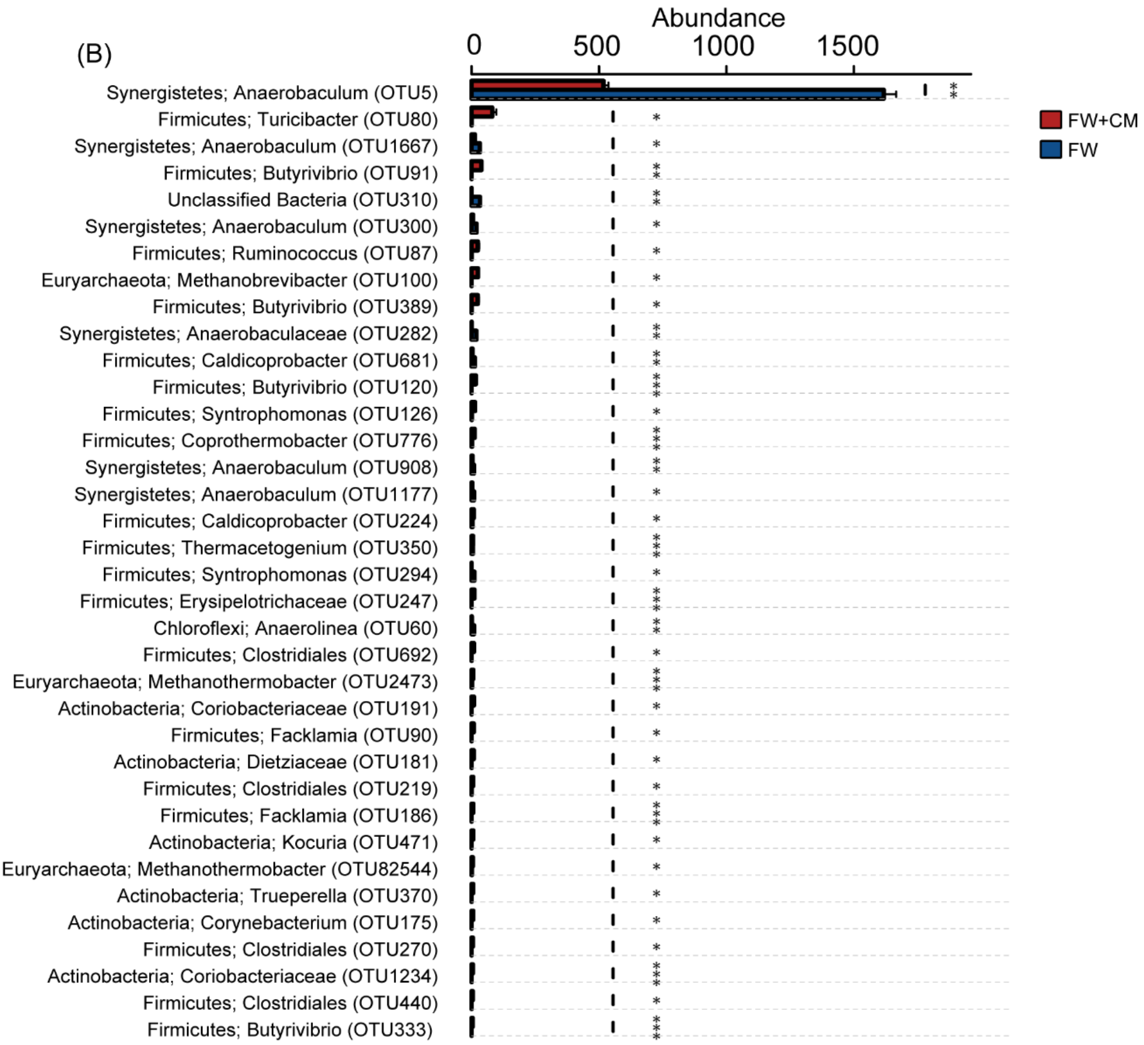

Figure S2: Anova comparison of taxa abundance in the microbial communities in the co-digesters (FW+CM; Red) and digester fed food waste only (FW; Blue), during both mesopilic (A) and thermophilic (B) processes. Standars deviations (initial vs. final) are included as error bars. The comparison are performed at OTU level, in which the OTUs are classified at genus or highest possible ranked taxonomic level. A comprehensive OTU table is provided as Supplementary Table S1 (separate material). Note that only significantly different OTUs are showed in the barchart, and annotated as *: p<0.005, **: p<0.01, ***:p<0.001.
